# Association of Daily Snow Depth with Emergency Medical Services Response and Survival After Out-of-Hospital Cardiac Arrest: A Prefectural Cohort Study in Northern Japan

**DOI:** 10.3390/jcm15145620

**Published:** 2026-07-17

**Authors:** Kyohei Maeno, Kasumi Satoh, Manabu Okuyama, Hajime Nakae

**Affiliations:** 1 Advanced Emergency and Critical Care Center, Akita University Hospital, Akita 010-0041, Japan; kyoro.m@gmail.com; 2 Department of Emergency and Critical Care Medicine, Akita University Graduate School of Medicine, Akita 010-8543, Japan; okuyamanabu@med.akita-u.ac.jp (M.O.); nakaeh@doc.med.akita-u.ac.jp (H.N.)

**Keywords:** out-of-hospital cardiac arrest, emergency medical services, snow, ambulance response time, treatment outcome, retrospective studies, cohort studies

## Abstract

**Background/Objectives:** Snow can disrupt emergency medical services (EMSs); however, previous studies have mainly measured snowfall or prefecture-level exposure. These measures may not capture snow remaining on the ground or conditions within ambulance operating areas. We examined whether the daily snow depth assigned at the fire department level was associated with EMS time intervals and 1-month survival after out-of-hospital cardiac arrest (OHCA). **Methods:** This retrospective cohort study included 7395 adults with OHCA from the Akita Prefecture Utstein-style emergency transport registry between 2019 and 2023. Daily snow depth from the nearest Automated Meteorological Data Acquisition System (AMeDAS) station was assigned to each case by the fire department. Snow exposure was analyzed as >0 cm versus 0 cm, as five depth categories, and as a continuous variable using natural splines. Multivariable models were adjusted for age, sex, cardiac origin, initial rhythm, fire department area, witnessed status, bystander cardiopulmonary resuscitation, and year. **Results:** Call-to-scene time and total EMS time were longer with snow cover than without snow cover (median, 9 vs. 8 min and 34 vs. 31 min, respectively; both *p* < 0.001). Snow cover was associated with lower 1-month survival after adjustment (odds ratio [OR], 0.73; 95% confidence interval [CI], 0.54–0.98), but this association was attenuated after additional adjustment for call-to-scene time (OR, 0.77; 95% CI, 0.57–1.03). Category-based and spline analyses showed no clear dose–response relationship. **Conclusions:** Daily snow depth is consistently associated with longer EMS response and transport times. However, its association with 1-month survival remains unclear. This survival association may reflect broader winter conditions rather than snow cover itself.

## 1. Introduction

Out-of-hospital cardiac arrest (OHCA) is a time-dependent condition, and the intervals from cardiac arrest to emergency medical service (EMS) arrival and hospital arrival are related to outcomes [1,2]. Therefore, identifying the factors that prolong EMS time intervals is a public health priority for improving emergency care systems. In snow-prone regions, road conditions can deteriorate during the winter, potentially delaying ambulance arrival at the scene. However, the effects of snow cover on EMS time intervals and outcomes after OHCA have not been sufficiently clarified.

Several studies have examined the associations between season, weather conditions, and OHCA; however, detailed evidence on snow exposure remains limited. Cold temperatures and winter conditions are associated with an increased risk of OHCA [3,4], lower survival, and poorer neurological outcomes [5,6]. Rainfall and heavy precipitation have also been reported to prolong ambulance response times [7,8]. Regarding snow exposure, nationwide registry studies in Japan have examined EMS response intervals among patients with OHCA [9,10]. However, many previous studies used temperature, season, precipitation, or snowfall as exposures, none of which directly measure the snow remaining on the ground. In contrast to snowfall, which represents newly fallen snow over a defined period, snow depth represents the depth of snow cover remaining on the ground and may be more closely related to driving conditions and road accessibility encountered by ambulances.

Two nationwide Japanese registry studies are particularly relevant. Omatsu et al. [9] compared patients with OHCA according to combinations of heavy snowfall area status and winter season using the All-Japan Utstein Registry. They found that EMS response intervals were longer in areas with heavy snowfall during winter, whereas the effects on 1-month survival and neurological outcomes were limited. Wataki et al. [10] used prefecture-level daily snowfall as the exposure among patients with cardiogenic OHCA and showed that ambulance response times were prolonged on heavy snowfall days; however, they did not evaluate patient outcomes. These studies have left several important issues unresolved. First, the designation as a heavy snowfall area is a fixed administrative classification and cannot capture daily variation. Second, snowfall indicates newly fallen snow but does not directly reflect the amount of snow remaining on roads. Third, broad geographic units, such as municipalities or prefectures, may not correspond to the operational units used for EMS transport, such as fire department areas. Fourth, the extent to which transportation delays contribute to worse outcomes remains unclear.

The objective of this study was to examine the association of daily snow depth assigned to each OHCA case at the fire department area level using the Akita Prefecture Utstein-style emergency transport registry with 1-month survival and EMS time intervals.

## 2. Materials and Methods

### 2.1. Study Design and Setting

This retrospective cohort study used data from the Akita Prefecture emergency transport registry, following the Utstein reporting template [2]. Akita Prefecture is located on the Sea of Japan side of northern Honshu, Japan, and has a population of approximately 900,000 in 2023, making it one of the most aged populations in Japan [11]. The entire prefecture has been designated as a heavy-snowfall area under the Act on Special Measures Concerning Heavy Snowfall Areas [12]. Akita Prefecture has 13 fire departments, which are listed in Supplementary Table S1. The registry includes all transported OHCA cases managed by all fire departments in the prefecture. The study period was 5 years, from 1 January 2019 to 31 December 2023. This study was approved by the Ethics Committee of the Akita Prefecture Medical Control Council (approval number: 0013). This study was conducted in accordance with the Declaration of Helsinki and the Ethical Guidelines for Life Science and Medical Research Involving Human Subjects in Japan. Because this was a retrospective analysis of existing registry data, the requirement for individual informed consent was waived through an opt-out procedure.

### 2.2. Participants

We included all patients aged 18 years or older with OHCA who were transported within Akita Prefecture during the study period. Patients younger than 18 years and those with inconsistent major outcome data, defined as survival at 1 month despite being recorded as Cerebral Performance Category (CPC) 5, were excluded. No formal sample size calculation was performed. To maximize the statistical power, all eligible cases during the 5-year study period were included. Because of the structure of the emergency transport registry, vital status at 1 month was confirmed for all cases, and no patients were lost to follow-up.

### 2.3. Data Sources and Data Management

Data on OHCA patients were obtained from the Akita Prefecture emergency transport registry, which is based on the Utstein template. Data were collected from each fire department through the Akita Prefecture Medical Control Council. The following clinical data were extracted: date of event; event location, defined by fire department area; patient characteristics, including age and sex; arrest characteristics, including witnessed status, cardiac origin, and initial rhythm (ventricular fibrillation [VF], pulseless ventricular tachycardia [pVT], pulseless electrical activity [PEA], asystole, or other); bystander interventions, including cardiopulmonary resuscitation (CPR), chest compression, ventilation, and automated external defibrillator (AED) use; EMS interventions, including defib-rillation, airway management, intravenous access, and adrenaline administration; EMS timestamps, including emergency call, scene arrival, patient contact, scene departure, and hospital arrival; and outcomes, including prehospital return of spontaneous circulation (ROSC), 1-month survival, and CPC at 1 month.

Snow-depth data were obtained from daily snow depth observations using the Japan Meteorological Agency Automated Meteorological Data Acquisition System (AMeDAS). AMeDAS is an automated meteorological observation system operated by the Japan Meteorological Agency. Snow depth is recorded in 1 cm units as the height from the ground surface to the snow surface [13]. Snow-depth observation stations in Akita Prefecture were used, and their correspondence with fire department areas is shown in Supplementary Table S1.

### 2.4. Exposure

The exposure variable was the daily snow depth at the location of cardiac arrest on the date of occurrence. In the primary analysis, snow cover was defined as a binary variable: snow cover was >0 cm, and no snow cover was 0 cm. Snow depth was chosen as the primary exposure because, whereas snowfall indicates the amount of newly fallen snow over a defined period, snow depth reflects the snow cover remaining on the ground and may therefore be more closely related to road conditions and vehicle mobility.

Each fire department area was prespecified to correspond to the nearest AMeDAS snow depth observation station based on geographic distance. The daily snow depth recorded at the corresponding station on the event date was assigned to each case. The correspondence between fire department areas and observation stations is provided in  Supplementary Table S1.

Snow depth was analyzed in three ways. First, the primary analysis used a binary classification of snow cover (>0 cm vs. 0 cm). Second, a category-based analysis classified snow depth into five groups: 0, 1–10, 11–30, 31–70, and >70 cm. The cut-offs were based on the quartiles of snow depth among cases with snow cover: first quartile, 10 cm; median, 30 cm; and third quartile, 69 cm. Third, among the cases with snow cover, snow depth was modeled as a continuous variable using natural splines to examine potential nonlinear associations.

### 2.5. Outcomes

The primary outcome was survival 1 month after cardiac arrest. The secondary outcomes were EMS time intervals, with an emphasis on call-to-scene and total EMS times. A favorable neurological outcome was used for descriptive comparison and was defined as CPC 1 or 2 at 1 month, in accordance with the Utstein reporting convention [2].

### 2.6. Covariates and Missing Data

Covariates for the multivariable models were prespecified based on patient characteristics, cardiac arrest characteristics, regional differences, and calendar year, all of which could confound the association between snow cover and outcomes. Witnessed status, bystander CPR, and initial rhythm have been reported as major prognostic factors for OHCA outcomes [14]. Age, sex, cause of cardiac arrest, initial rhythm, witnessed status, bystander CPR, and outcomes were defined according to standardized OHCA registry items in the Utstein reporting convention [2]. The multivariable models included age, sex, cardiac origin, initial rhythm, fire department area, witness status, bystander CPR, and year of occurrence. In the primary analysis, the initial rhythm was classified as shockable (VF/pVT) or nonshockable. A sensitivity analysis was performed using a model that included individual initial rhythm categories (VF, pVT, PEA, asystole, and others; Supplementary Table S2). Fire department area was included as a set of dummy variables to adjust for regional differences in transport conditions related to catchment area, population density, and medical resources. The year of occurrence was included as a continuous variable to account for temporal changes in EMS systems and OHCA care, including changes in bystander intervention and public access defibrillation during the COVID-19 pandemic [15].

The EMS time intervals were calculated in minutes from the timestamps of the Utstein data. The intervals were call-to-scene time, defined as the interval from emergency call to scene arrival; scene time, defined as the interval from scene arrival to scene departure; scene-to-hospital time, defined as the interval from scene arrival to hospital arrival; and total EMS time, defined as the interval from emergency call to hospital arrival.

The primary outcome and all covariates included in the multivariable models had no missing data, and multiple imputation was not used. Bystander AED use was excluded from the multivariable models because of substantial missing data. The missing variables used or considered in the analyses are listed in Supplementary Table S3.

### 2.7. Statistical Analysis

The analysis proceeded in three steps.

First, the patient characteristics were compared between the snow cover group (>0 cm) and the no-snow group (0 cm; Table 1). Continuous variables were described as medians with interquartile ranges and compared using the Mann–Whitney U test. Categorical variables were described as counts and percentages and compared using the chi-square test.

Second, the association between snow cover and 1-month survival was assessed using a multivariable logistic regression with sequential adjustments. Sequential adjustment was designed to describe how the estimated association between snow cover and 1-month survival changed after adjusting for call-to-scene time. Model 1 was unadjusted and included only snow cover status. Model 2 was adjusted for age, sex, cardiac origin, initial rhythm (shockable vs. non-shockable), fire department area, witness status, bystander CPR, and year of occurrence. Model 3 also included call-to-scene time to explore changes in the estimated association. The results are reported as odds ratios (ORs) with 95% confidence intervals (CIs). The change in the estimate from Model 2 to Model 3 was interpreted descriptively with reference to the change-in-estimate approach [16]; this analysis was not intended as a formal mediation analysis.

Third, EMS time intervals and survival were examined across snow depth categories (Table 3 and Figure 2). Using the categories defined above (0, 1–10, 11–30, 31–70, and >70 cm), adjusted mean differences in call-to-scene time and total EMS time relative to 0 cm were estimated using multivariable linear regression, and adjusted ORs for 1-month survival were estimated using multivariable logistic regression. The adjustment variables are identical to those in Model 2 in Table 2. Among the cases with snow cover, snow depth was also modeled as a continuous variable using natural splines with three degrees of freedom to examine nonlinear associations (Figure 3). Nonlinearity was assessed using likelihood ratio tests by comparing the spline and linear models.We performed four sensitivity analyses. First, the initial rhythm was included as a five-level variable (VF, pVT, PEA, asystole, etc.) to assess whether the point estimates were consistent with those from the main binary shockable rhythm classification. Second, to assess the influence of seasonal confounding factors, the analysis was restricted to the winter months (December to February), and the association between snow cover and 1-month survival was re-estimated. This winter-restricted analysis examined the direction of the association under conditions in which seasonal confounding was partly reduced. Because of the limited number of events, the full 13-level fire department area was excluded; as a further analysis, the areas were instead grouped into a two-level coastal/inland region block and re-entered as a covariate. The remaining covariates were identical to those in the primary analysis. Third, the binary snow depth cut-off was changed to >10, >30, >50, and >70 cm to assess the stability of the direction and magnitude of the ORs. Fourth, a pandemic-period indicator (2019 vs. 2020–2023) and, separately, month (as a categorical variable) were added to Model 2 to assess temporal and seasonal confounding. In both analyses, the continuous calendar-year term in Model 2 was retained; the pandemic-period indicator therefore captured a level shift between the pre-pandemic and pandemic periods beyond the linear secular trend modeled by calendar year. The sensitivity analyses are summarized in Supplementary Table S2.

All statistical analyses were performed using R version 4.3.3 (R Foundation for Statistical Computing, Vienna, Austria). The gtsummary package (version 2.3.0) was used to generate descriptive statistics and regression tables and the splines package (version 4.3.3) was used to implement natural splines. All tests were two-sided, and *p* < 0.05 was considered statistically significant.

## 3. Results

### 3.1. Participant Selection

**Figure 1:**
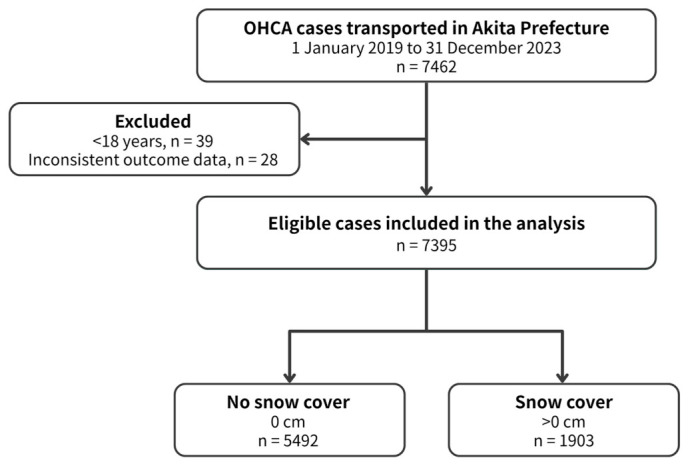
**Study flow diagram.** OHCA, out-of-hospital cardiac arrest.

During the study period, 7462 patients with OHCA were transported in Akita Prefecture. After excluding 39 patients aged < 18 years and 28 patients with inconsistent outcome data, 7395 patients were included in the final analysis (Figure 1). The snow cover group (snow depth > 0 cm) included 1903 patients (25.7%) and the no-snow group (0 cm) included 5492 patients (74.3%). Among cases with snow cover, the median snow depth was 30 cm (interquartile range, 10–69). The distribution by snow-depth category was 5492 cases (74.3%) for 0 cm, 496 (6.7%) for 1–10 cm, 456 (6.2%) for 11–30 cm, 486 (6.6%) for 31–70 cm, and 465 (6.3%) for >70 cm; the snow-cover categories were therefore approximately balanced. The monthly distributions of OHCA cases and mean snow depths are shown in Supplementary Figure S1.

### 3.2. Patient Characteristics

The cohort consisted of 7395 patients (Table 1). The median age was 83 years (interquartile range, 73–89), 4025 patients (54.4%) were male, and 4241 cases (57.3%) were of cardiac origin. Arrest was witnessed in 3096 patients (41.9%), bystander CPR was performed in 4352 patients (58.9%), and the initial rhythm was shockable (VF/pVT) in 375 patients (5.1%). One-month survival occurred in 313 patients (4.2%), and CPC 1–2 at 1 month were recorded in 168 patients (2.3%).

Compared to the no-snow group, the snow-cover group had significantly longer EMS time intervals. The median call-to-scene time was 9 min (interquartile range, 7–12) in the snow cover group and 8 min (6–11) in the no-snow group (*p* < 0.001). The median scene time was also longer in the snow cover group (11 min [9–13] vs. 10 min [8–12]; *p* < 0.001). Scene-to-hospital time (24 min [19–32] vs. 22 min [17–31]; *p* < 0.001) and total EMS time (34 min [27–43] vs. 31 min [25–41]; *p* < 0.001) were similarly prolonged in the snow-cover group.

One-month survival was significantly lower in the snow-cover group than in the no-snow group (3.4% [64/1903] vs. 4.5% [249/5492], *p* = 0.029). No significant differences were observed in prehospital ROSC (7.0% vs. 7.7%; *p* = 0.321) or CPC 1–2 at 1 month (1.9% vs. 2.4%; *p* = 0.306).

### 3.3. Association Between Snow Cover and 1-Month Survival

This analysis of 1-month survival, the prespecified primary outcome, was exploratory. Table 2 shows the results of the sequential multivariable logistic regression. In the unadjusted model (Model 1), the OR for 1-month survival in the snow-cover group was 0.73 (95% CI, 0.55–0.96; *p* = 0.029). In Model 2, adjusted for age, sex, cardiac origin, initial rhythm, fire department area, witnessed status, bystander CPR, and year of occurrence, the OR was 0.73 (95% CI, 0.54–0.98; *p* = 0.037). In Model 3, which additionally included call-to-scene time, the OR was attenuated to 0.77 (95% CI, 0.57–1.03; *p* = 0.085), and the 95% CI crossed 1.0.

### 3.4. EMS Time Intervals and Survival by Snow-Depth Category

**Figure 2:**
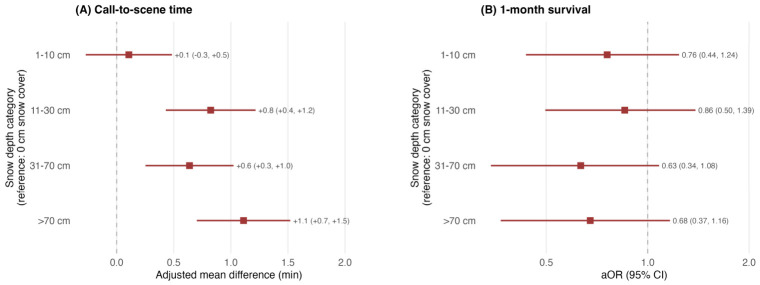
**EMS time intervals and 1-month survival by snow-depth category.** Adjusted estimates relative to 0 cm are shown for (**A**) call-to-scene time and (**B**) 1-month survival. Points indicate point estimates, and horizontal lines indicate 95% CIs. Models were adjusted for age, sex, cardiac origin, initial rhythm (shockable vs. non-shockable), fire department area, witnessed status, bystander CPR, and year of occurrence. Notes: aOR, adjusted odds ratio.

The adjusted estimates for EMS time intervals and 1-month survival by snow depth category are shown in Table 3 and Figure 2.

For EMS time intervals, moderate and high snow depth categories were associated with longer call-to-scene times. Compared with 0 cm, the adjusted mean difference in call-to-scene time was +0.11 min for 1–10 cm (*p* = 0.580), +0.82 min for 11–30 cm (*p* < 0.001), +0.64 min for 31–70 cm (*p* = 0.001), and +1.11 min for >70 cm (*p* < 0.001). The total EMS time showed a similar pattern, with a +4.01 min prolongation in the >70 cm category (*p* < 0.001).

Adjusted ORs for 1-month survival were below 1.0 in all categories, but no clear dose–response relationship was observed, and none of the category-specific estimates reached statistical significance (1–10 cm: OR, 0.76; 95% CI, 0.44–1.24; 11–30 cm: OR, 0.86; 95% CI, 0.50–1.39; 31–70 cm: OR, 0.63; 95% CI, 0.34–1.08; >70 cm: OR, 0.68; 95% CI, 0.37–1.16).

### 3.5. Nonlinear Associations Between Snow Depth and Outcomes

**Figure 3:**
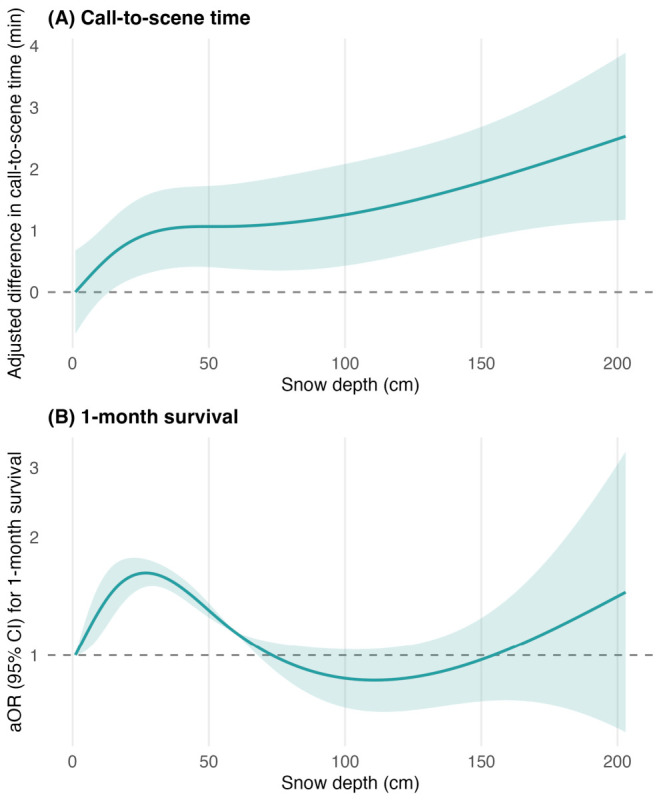
**Nonlinear associations of snow depth with EMS time intervals and 1-month survival using natural splines.** Among cases with snow cover (>0 cm, n = 1903), snow depth was modeled as a continuous variable using natural splines with 3 degrees of freedom. (**A**) Adjusted difference in call-to-scene time (min). (**B**) OR for 1-month survival with 95% CI. Snow depth of 1 cm was used as the reference. Solid lines indicate point estimates, and shaded areas indicate 95% CIs. Nonlinearity was assessed using likelihood ratio tests (call-to-scene time, *p* = 0.148; survival, *p* = 0.453). Models were adjusted for age, sex, cardiac origin, initial rhythm (shockable vs. non-shockable), fire department area, witnessed status, bystander CPR, and year of occurrence. Panel B (1-month survival) is exploratory: only 241 cases (7 events) had snow depths above 100 cm, where the estimates are unstable.

Among the cases with snow cover (n = 1903), snow depth was modeled as a continuous variable using natural splines (Figure 3). For call-to-scene time, the test for nonlinearity was not significant (*p* = 0.148). For 1-month survival, the test for nonlinearity was also not significant (*p* = 0.453), although the spline curve showed visual fluctuations in the high snow depth range. Only 241 patients had snow depths greater than 100 cm, with seven outcome events, suggesting that sparse data may have contributed to the instability of the estimates in this range.

### 3.6. Sensitivity Analyses

The sensitivity analyses are summarized in Supplementary Table S2. When initial rhythm was modeled using individual categories, the OR for snow cover was 0.78 (95% CI, 0.57–1.06; *p* = 0.124), which was directionally consistent with the primary analysis using the binary shockable-rhythm classification. In the winter-restricted adjusted analysis (December-February; n = 2187; snow-cover group, n = 1622; no-snow group, n = 565; events, n = 79), the adjusted OR was 0.87 (95% CI, 0.52–1.49; *p* = 0.589). Although this estimate was not statistically significant, its direction was consistent with that of the primary analysis. With the coastal/inland region block re-entered, the adjusted OR was 0.89 (95% CI, 0.52–1.57). Adding a pandemic-period indicator or month to Model 2 yielded ORs of 0.74 (95% CI, 0.55–0.99) and 0.83 (95% CI, 0.52–1.31), respectively. When the binary snow depth cutoff was changed to >10, >30, >50, and >70 cm, the ORs were below 1.0, which is consistent with the direction of the primary analysis.

## 4. Discussion

### 4.1. Key Findings and Strengths

To our knowledge, this is the first study to assign daily snow depth to individual OHCA cases at the fire department level in a snow-prone region of Japan and to examine its association with both EMS time intervals and 1-month survival. The main findings are as follows. First, the snow-cover group had longer call-to-scene, scene, scene-to-hospital, and total EMS times. Second, category-based analyses showed longer EMS time intervals in the moderate and high snow depth categories. Third, the adjusted point estimate for 1-month survival was below 1.0; however, the estimate was attenuated after additional adjustment for call-to-scene time, and the 95% CI crossed 1.0. Fourth, category-based and spline analyses did not show a clear dose–response relationship for 1-month survival.

This study addresses two limitations of the previous work [9,10]. First, rather than using snowfall or administrative heavy snowfall area designations, we used daily snow depth as the exposure, because it is likely to reflect road surface conditions more directly. Second, by linking each fire department area to the nearest AMeDAS observation station and assigning daily snow depth to individual cases by event date, this study achieved finer temporal and spatial resolutions than previous studies.

### 4.2. Interpretation

Using snow depth as the main exposure extended earlier studies that used the administrative heavy-snowfall area status or daily snowfall [9,10]. Whereas snowfall reflects newly fallen snow, snow depth reflects snow remaining on the ground, and may therefore better approximate the driving environment that ambulances face. The observed association between snow cover and longer EMS time intervals was the main and most robust finding of this study.

Moreover, snow-cover status is strongly correlated with season. Therefore, this study could not completely separate the effects of snow cover from the broader effects of winter conditions. In the winter-restricted sensitivity analysis, the direction of the association was consistent with that of the primary analysis and did not materially undermine the main interpretation; however, the estimate was imprecise. Therefore, the findings for 1-month survival should be interpreted cautiously, not as evidence of an independent effect of snow depth itself but rather as an association that may more broadly reflect adverse environmental conditions accompanying snow cover or winter conditions. The association persisted after a pandemic-period indicator was added (OR, 0.74) but attenuated when within-season timing was accounted for, whether by restricting to winter (OR, ~0.89) or adjusting for month (OR, 0.83), indicating that part of it reflects seasonal timing rather than snow cover itself. We therefore regard the 1-month survival finding as exploratory.

In Model 3, which also included call-to-scene time, the association between snow cover and 1-month survival was modestly attenuated, although the point estimate remained below 1.0. This finding is consistent with the possibility that call-to-scene time contributed to the observed association. However, the 95% CI crossed 1.0, and the sequential adjustment used in this study was not a formal mediation analysis. Therefore, it is not possible to conclude that transport delays partially explain this association; this comparison is descriptive and does not establish mediation. In patients with acute coronary syndrome, snowfall days were associated with longer EMS transport times and higher in-hospital mortality [17]. This indirectly suggests that transport delay under snowy conditions may influence outcomes in acute cardiovascular disease; however, the relationship between snow depth, EMS delay, and outcomes after OHCA requires a more detailed evaluation.

### 4.3. Clinical Implications

These findings support the need for winter preparedness in EMS systems serving snow-prone regions. During snow cover, delays were observed not only in call-to-scene time but also in scene time and time to hospital arrival. These delays may reflect deteriorated driving conditions, difficulty in accessing the scene, constraints on patient extrication routes, and delays in patient discovery or emergency calls. In snowy conditions, ambulances may be unable to park immediately adjacent to the incident location, and EMS personnel may need to walk over snow while carrying equipment. Winter conditions may also influence the processes before EMS activation, including patient discovery and emergency call timing, as well as differences in the circumstances of arrest under cold conditions. In snow-prone regions, optimizing winter ambulance deployment, coordinating snow clearance operations, and reviewing field access systems, including physician-staffed rapid-response vehicles, where available, may help reduce EMS delays. Whether these operational improvements translate into improved clinical outcomes requires further investigation.

### 4.4. Limitations

This study had some limitations. First, as this was a retrospective observational study, causality could not be established. Second, snow-cover status is strongly correlated with season, and residual confounding from temperature, daylight, infectious disease activity, and winter-specific case mix could not be fully eliminated. Therefore, the findings should not be interpreted as a pure estimate of the snow-cover effects. Third, assigning a value from the nearest observation station to each fire department area did not capture local variations in snow depth, road icing, snow removal status, or differences in the timing of snowfall within an area, and exposure misclassification is possible. Fourth, the daily snow depth is a representative value for a day and does not directly capture short-term road conditions around the time of arrest. Fifth, sequential adjustment, including call-to-scene time, was not a formal mediation analysis and could not causally quantify any mediating effect of transport delays. Sixth, witnessed status and bystander CPR were treated as confounders; however, they could theoretically lie on the causal pathway between snow conditions and outcomes. Adjustment for such intermediate variables may have led to overadjustment and underestimation of the overall association [18]. Seventh, bystander AED use had substantial missing data and was not included in the multivariable models, leaving the possibility of residual confounding factors. Eighth, this was a single-prefecture study; therefore, the generalizability of the findings to other snow-prone regions may be limited. Ninth, the survival spline was based on few patients (n = 241) and events (n = 7) at snow depths above 100 cm, so the estimates in this range are unstable.

## 5. Conclusions

In this cohort study of patients with OHCA in Akita Prefecture, daily snow depth was associated with delayed EMS scene arrival and longer overall transport time. The main contribution of this study is that these EMS delays were consistently observed even when the daily snow depth was assigned at the fire department area level. In contrast, the analysis of 1-month survival, the prespecified primary outcome, was exploratory; the observed association may reflect broader winter conditions rather than snow cover itself and requires further validation.

## Figures and Tables

**Table 1:** **Patient characteristics according to snow-cover status.** Patients with OHCA in Akita Prefecture were classified into the snow-cover group (snow depth >0 cm, n = 1903) and the no-snow group (0 cm, n = 5492). Continuous variables are shown as medians [interquartile ranges], and categorical variables as n (%).

Characteristic	Overall, N = 7395	No Snow, N = 5492	Snow, N = 1903	*p*-Value
Age	83 [73, 89]	83 [73, 89]	84 [74, 89]	0.6
Male	4025 (54%)	3001 (55%)	1024 (54%)	0.5
Cardiac origin	4241 (57%)	3083 (56%)	1158 (61%)	<0.001
Witnessed arrest	3096 (42%)	2306 (42%)	790 (42%)	0.7
Bystander CPR (chest compression)	4352 (59%)	3205 (58%)	1147 (60%)	0.14
Bystander CPR (ventilation)	220 (3.4%)	158 (3.3%)	62 (3.7%)	0.5
Bystander AED	638 (10%)	490 (11%)	148 (9.2%)	0.092
Shockable rhythm (VF/pVT)	375 (5.1%)	286 (5.2%)	89 (4.7%)	0.4
EMS defibrillation	555 (10%)	413 (10%)	142 (10.0%)	>0.9
IV access	3879 (59%)	2852 (59%)	1027 (60%)	0.3
Adrenaline	1554 (27%)	1136 (27%)	418 (28%)	0.3
Call-to-scene time (min)	8.0 [7.0, 11.0]	8.0 [6.0, 11.0]	9.0 [7.0, 12.0]	<0.001
Scene time (min)	10.0 [8.0, 13.0]	10.0 [8.0, 12.0]	11.0 [9.0, 13.0]	<0.001
Scene-to-hospital time (min)	23 [17, 31]	22 [17, 31]	24 [19, 32]	<0.001
Total EMS time (min)	32 [25, 41]	31 [25, 41]	34 [27, 43]	<0.001
Snow depth (cm)	0 [0, 1]	0 [0, 0]	30 [10, 69]	<0.001
Snow-depth category				<0.001
0 cm	5492 (74%)	5492 (100%)	0 (0%)	
1–10 cm	496 (6.7%)	0 (0%)	496 (26%)	
11–30 cm	456 (6.2%)	0 (0%)	456 (24%)	
31–70 cm	486 (6.6%)	0 (0%)	486 (26%)	
>70 cm	465 (6.3%)	0 (0%)	465 (24%)	
Prehospital ROSC	557 (7.5%)	424 (7.7%)	133 (7.0%)	0.3
1-month survival	313 (4.2%)	249 (4.5%)	64 (3.4%)	0.029
CPC 1–2	168 (2.3%)	131 (2.4%)	37 (1.9%)	0.3

CPR, cardiopulmonary resuscitation; AED, automated external defibrillator; VF, ventricular fibrillation; pVT, pulseless ventricular tachycardia; EMS, emergency medical services; IV, intravenous; ROSC, return of spontaneous circulation; CPC, Cerebral Performance Category. *p*-values were calculated using the Wilcoxon rank-sum test for continuous variables and Pearson’s chi-squared test for categorical variables.

**Table 2:** **Association between snow cover and 1-month survival: sequential adjustment models.** The association between snow-cover status (>0 cm vs. 0 cm) and 1-month survival was assessed using multivariable logistic regression. Model 1 was unadjusted. Model 2 was adjusted for age, sex, cardiac origin, initial rhythm (shockable vs. non-shockable), fire department area, witnessed status, bystander CPR, and year of occurrence. Model 3 additionally included call-to-scene time. Results are shown as ORs with 95% CIs.

Characteristic	Model 1			Model 2			Model 3		
	OR	95% CI	*p*-Value	OR	95% CI	*p*-Value	OR	95% CI	*p*-Value
No snow (reference)	—	—	—	—	—	—	—	—	—
Snow	0.73	0.55, 0.96	0.029	0.73	0.54, 0.98	0.037	0.77	0.57, 1.03	0.085

Notes: OR, odds ratio; CI, confidence interval.

**Table 3:** **EMS time intervals and 1-month survival by snow-depth category.** For each snow-depth category (0, 1–10, 11–30, 31–70, and >70 cm), adjusted mean differences in call-to-scene time and total EMS time relative to 0 cm were estimated using multivariable linear regression, and adjusted ORs for 1-month survival were estimated using multivariable logistic regression. Cutoffs were prespecified based on quartiles among cases with snow cover. Models were adjusted for age, sex, cardiac origin, initial rhythm (shockable vs. non-shockable), fire department area, witnessed status, bystander CPR, and year of occurrence.

Snow Depth	Call-to-Scene Time (min)			Total EMS Time (min)			1-Month Survival		
	Beta	95% CI	*p*-Value	Beta	95% CI	*p*-Value	OR	95% CI	*p*-Value
0 cm (reference)	—	—	—	—	—	—	—	—	—
1–10 cm	0.11	−0.27, 0.48	0.6	0.39	−0.76, 1.6	0.5	0.76	0.44, 1.24	0.3
11–30 cm	0.82	0.43, 1.2	<0.001	2.7	1.5, 3.9	<0.001	0.86	0.50, 1.39	0.6
31–70 cm	0.64	0.25, 1.0	0.001	2.0	0.86, 3.2	<0.001	0.63	0.34, 1.08	0.12
>70 cm	1.1	0.70, 1.5	<0.001	4.0	2.8, 5.3	<0.001	0.68	0.37, 1.16	0.2

EMS, emergency medical services; CPR, cardiopulmonary resuscitation; OR, odds ratio; CI, confidence interval.

## Data Availability

The data analyzed in this study are not publicly available because they contain sensitive patient-level information from the Akita Prefecture emergency medical services registry and are subject to administrative and ethical restrictions. Data may be made available from the Akita Prefecture Medical Control Council upon reasonable request, with appropriate institutional and ethical approval.

## Supplementary Materials

The following supporting information can be downloaded at: https://www.mdpi.com/article/10.3390/jcm15145620/s1, Table S1: Correspondence between fire department areas and AMeDAS snow-depth observation stations; Table S2: The table summarizes sensitivity analyses for the association between snow cover and 1-month survival; Table S3. Missingness of variables used or considered in the analyses; Figure S1: Monthly number of OHCA cases and mean snow depth.

## Abbreviations

The following abbreviations are used in this manuscript:

AED	automated external defibrillator
AMeDAS	Automated Meteorological Data Acquisition System
CPC	Cerebral Performance Category
CPR	cardiopulmonary resuscitation
EMS	emergency medical services
OHCA	out-of-hospital cardiac arrest
OR	odds ratio
PEA	pulseless electrical activity
pVT	pulseless ventricular tachycardia
ROSC	return of spontaneous circulation
VF	ventricular fibrillation
