# Supplementary material for: Association of Daily Snow Depth with Emergency Medical Services Response and Survival After Out-of-Hospital Cardiac Arrest: A Prefectural Cohort Study in Northern Japan
Source: J Clin Med. 2026 Jul 17;15(14):5620. doi: 10.3390/jcm15145620 (PMC13412232; doi:10.3390/jcm15145620)
Supplement: Supplementary file 1 [file jcm-15-05620-s001.zip › jcm-4417696-supplementary.pdf]

Supplementary Materials

Table S1. Correspondence between fire department areas and AMeDAS snow-depth observation stations. The table lists the 13 fire department areas in Akita Prefecture and the corresponding AMeDAS snow-depth observation stations. Each fire department area was linked in advance to the geographically nearest observation station, and daily snow depth on the event date was assigned to each case. The number of cases (n) is based on the 7,395 eligible cases during the study period from 2019 to 2023. Akita Prefecture has 14 AMeDAS snow-depth observation stations (Noshiro, Takanosu, Kazuno, Gojome, Akita, Kakunodate, Honjo, Yokote, Yashima, Yuzawa, Yunotai, Daishoji, Aniai, and Yuwa). The present study used the eight stations listed below to assign snow depth to fire department areas.

Fire department area	AMeDAS station	n
Akita	Akita	1,680
Daisen	Yokote	1,179
Yokote	Yokote	846
Noshiro	Noshiro	626
Odate	Takanosu	601
Yurihonjo	Honjo	572
Yuzawa	Yuzawa	486
Oga	Gojome	391
Kazuno	Kazuno	331
Kita-Akita	Takanosu	303
Koto	Gojome	160
Nikaho	Honjo	152
Gojome	Gojome	68
Total		7,395

Table S2. The table summarizes sensitivity analyses for the association between snow cover and 1-month survival. All analyses used snow-cover status (>0 cm vs. 0 cm) as the main exposure and are compared with the primary analysis (Model 2 in Table 2).Adjustment variables: age, sex, cardiac origin, initial rhythm (binary shockable rhythm in the primary analysis and sensitivity analyses 3a-3d; five categories in sensitivity analysis 1), fire department area (excluded in sensitivity analysis 2), witnessed status, bystander CPR, and year of occurrence. In sensitivity analysis 2, fire department area was excluded because of the limited number of events in the winter-restricted analysis. In sensitivity analysis 2b, the 13 fire department areas were grouped into a coastal/inland region block and re-entered as a covariate. Sensitivity analysis 4 added a pandemic-period indicator (2019 vs. 2020-2023) and sensitivity analysis 4b added month, each to Model 2.

Analysis	N	Events	OR	95% CI	p-value
Primary analysis (Model 2, binary shockable rhythm)	7,395	313	0.73	0.54-0.98	0.037
Sensitivity analysis 1: initial rhythm modeled as five categories (VF/pVT/PEA/asystole/other)	7,395	313	0.78	0.57-1.06	0.124
Sensitivity analysis 2: restricted to winter (December-February), excluding fire department area	2,187	79	0.87	0.52-1.49	0.589
Sensitivity analysis 2b: winter (December-February) + coastal/inland region block	2,187	79	0.89	0.52-1.57	0.682
Sensitivity analysis 3a: cutoff >10 cm vs. <=10 cm	7,395	313	0.74	0.52-1.02	0.073
Sensitivity analysis 3b: cutoff >30 cm vs. <=30 cm	7,395	313	0.67	0.43-1.00	0.060
Sensitivity analysis 3c: cutoff >50 cm vs. <=50 cm	7,395	313	0.53	0.31-0.86	0.016
Sensitivity analysis 3d: cutoff >70 cm vs. <=70 cm	7,395	313	0.72	0.39-1.23	0.254
Sensitivity analysis 4: Model 2 + pandemic-period indicator (2019 vs. 2020-2023)	7,395	313	0.74	0.55-0.99	0.047
Sensitivity analysis 4b: Model 2 + month	7,395	313	0.83	0.52-1.31	0.419

Table S3. Missingness of variables used or considered in the analyses. The table shows missingness among the 7,395 eligible cases for variables used or considered in this study. Variables included in the multivariable models and the primary outcome had no missing data; therefore, multiple imputation was not used. Bystander AED use is listed separately because it had substantial missingness and was excluded from multivariable models.

Variable group	Missingness
Variables included in multivariable models (age, sex, cardiac origin, initial rhythm, witnessed status, bystander CPR, fire department area, year of occurrence, call-to-scene time, and total EMS time)	0%
Primary outcome (1-month survival)	0%
Secondary outcomes (prehospital ROSC and CPC at 1 month)	0%
Exposure variable (daily snow depth)	0%
Bystander AED use (excluded from multivariable models because of missingness)	37.8%

Figure S1. Monthly number of OHCA cases and mean snow depth

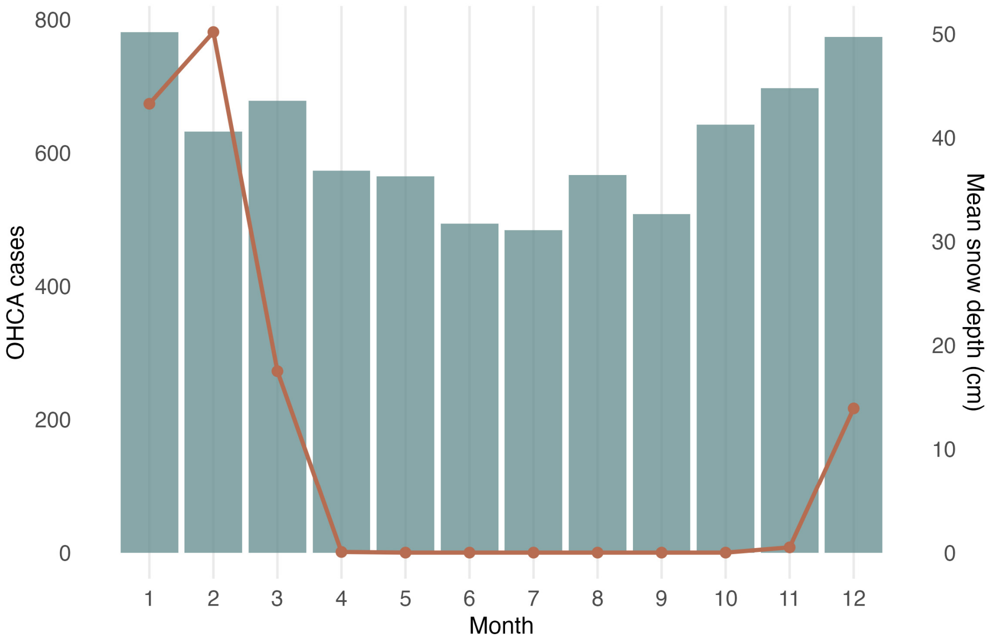

This figure shows the monthly number of OHCA cases and monthly mean daily snow depth at the event location among eligible cases during the study period from 2019 to 2023. Bars indicate monthly OHCA cases (teal bars and left axis) and the line indicates mean snow depth in centimeters (orange line and right axis).
